# Enhancing xanthine dehydrogenase activity is an effective way to delay leaf senescence and increase rice yield

**DOI:** 10.1186/s12284-020-00375-7

**Published:** 2020-03-11

**Authors:** Ruicai Han, Xunfeng He, Xiaohua Pan, Qinghua Shi, Ziming Wu

**Affiliations:** 1 Key Laboratory of Crop Physiology, Ecology and Genetic Breeding, Ministry of Education, College of Agronomy, Jiangxi Agricultural University, Nanchang, China; 2 Rice Research Institute, Jiangxi Academyof Agricultural Sciences/Jiangxi Provincial Key Laboratory for Physiology and Genetics of Rice, Nanchang, China

**Keywords:** Rice (*Oryza sativa* L.), Xanthine dehydrogenase, Allantoin, Reactive oxygen species, Senescence, Yield

## Abstract

Xanthine dehydrogenase (XDH) is an important enzyme in purine metabolism. It is involved in regulation of the normal growth and non-biological stress-induced ageing processes in plants. The present study investigated XDH’s role in regulating rice leaf senescence. We measured physical characteristics, chlorophyll content and fluorescence parameters, active oxygen metabolism, and purine metabolism in wild-type Kitaake rice (*Oryza sativa* L.), an *OsXDH* over-expression transgenic line (*OE9*), and an *OsXDH* RNA interference line (*Ri3*) during different growth stages. The expression patterns of the *OsXDH* gene confirmed that XDH was involved in the regulation of normal and abiotic stress-induced ageing processes in rice. There was no significant difference between the phenotypes of transgenic lines and wild type at the seedling stage, but differences were observed at the full heading and maturation stages. The *OE9* plants were taller, with higher chlorophyll content, and their photosystems had stronger light energy absorption, transmission, dissipation, and distribution capacity, which ultimately improved the seed setting rate and 1000-seed weight. The opposite effect was found in the *Ri3* plants. The *OE9* line had a strong ability to remove reactive oxygen species, with increased accumulation of allantoin and alantoate. Experimental spraying of allantoin on leaves showed that it could alleviate chlorophyll degradation and decrease the content of H_2_O_2_ and malonaldehyde (MDA) in rice leaves after the full heading stage. The urate oxidase gene (*UO*) expression levels in the interference line were significantly lower than those in the over-expression line and wild-type lines. The allantoinase (*ALN*) and allantoate amidinohydrolase (*AAH*) genes had significantly higher expression in the *Ri3* plants than the in *OE9* or wild-type plants, with *OE9* plants showing the lowest levels. The senescence-related genes *ACD1*, *WRKY23*, *WRKY53*, *SGR*, *XERO1*, and *GH27* in *Ri3* plants had the highest expression levels, followed by those in the wild-type plants, with *OE9* plants showing the lowest levels. These results suggest that enhanced activity of XDH can regulate the synthesis of urea-related substances, improve plant antioxidant capacity, effectively delay the ageing process in rice leaves, and increase rice yield.

## Introduction

Rice (*Oryza sativa* L.) is subject to premature senility during its late growth period, manifesting as premature deterioration of metabolic function, which seriously hinders the improvement of rice yield (Großkinsky et al. 2018). Leaves turn yellow prematurely and photosynthetic function declines significantly, which in turn affects the yield and quality of grains (Inada et al. 1999; Kim et al. 2011; Zhang et al. 2007). According to theoretical calculations, 60% to 80% of the nutrients required for grain filling after rice heading derive from leaf photosynthesis (Fageria 2007). In the later phase of rice grain filling, extending the growth of useful leaves by 1 d would result in a yield increase of approximately 1% to 2% (Zhu et al. 2012). Given these findings, improvement in photosynthetic activity in rice leaves could be key to reaching high or even super-high yields.

Recent studies conducted into the causes of plant senescence relating to leaf morphology and physiological characteristics have hypothesised contributory factors such as nutrient deficiency, plant hormones, reactive oxygen metabolism, death factors, accumulated genetic errors, photoperiod, and senescence genes (Becker and Apel 1993; Buchanan-Wollaston 1997; Gan and Amasino 1997; Khanna-Chopra 2012; Park et al. 2007; Peng and Peng 2000; Rajinder et al. 1981; Tang et al. 2005). Leaf senescence is found to be a complex physiological process affected by, for example, low chlorophyll (Buchanan-Wollaston 1997; Peng and Peng 2000), high malondialdehyde (MDA) content (Rajinder et al. 1981), reduced free radical and reactive oxygen scavenging enzyme activity, decomposition of nucleic acids, proteins and other macromolecules (Park et al. 2007), and nutrient reuse (Simpson and Dalling 1981).

Xanthine dehydrogenase (XDH), a molybdenum-containing hydroxylase, is a major enzyme regulating the metabolism of purines. Xanthine dehydrogenase can catalyze the formation of uric acid from xanthine and hypoxanthine, and then the formation of allantoin and allantoate through a series of metabolic reactions (Werner and Witte 2011). The mechanisms of XDH involvement in the regulation of ageing and stress resistance have been studied in Arabidopsis (Brychkova et al. 2008), pea (Zdunek-Zastocka and Lips 2003), corn (Katalin et al. 2000), and grapes (You et al. 2017). The regulation mechanism of XDH involved in plant leaf premature senility involves multiple processes such as metabolism of nitrogen (Hofmann 2016), reactive oxygen species (Ma et al. 2016), and hormones (Cowan and Taylor 2004). The XDH amino acid sequence of rice was similar to that of arabidopsis and corn by 69.12% and 76.35%, respectively, and XDH homology has been found to be relatively high among different species of advanced plants (Han et al. 2017). However, there are few studies on how XDH could delay the senescence of rice leaves. We therefore undertook this study, in which we evaluated the effects of overexpression and defects in XDH transcription on rice phenotype and physiology during different growth stages. Our aim was to determine the physiological mechanism of XDH-mediated delay in rice leaf senescence.

## Materials and Methods

### Plant Material and Growth Conditions

An *OsXDH* (LOC4333171) over-expression transgenic line (*OE9*), an RNA interference line (*Ri3*), and wild type rice Kitaake (*Oryza sativa* L.) were used for functional validation of XDH against premature senility in rice. The *OsXDH* full-length coding region was amplified and cloned into pCUBI1390 to generate pCUBI1390:*OsXDH* over-expression construct. To generate the *OsXDH*-RNAi vector, a specific sequence of the *OsXDH* coding region was amplified. The resulting PCR product was inserted into the LH-FAD2-1390RNAi vector in both sense and antisense orientation to generate LH-FAD2-1390RNAi:*OsXDH OsXDH*-RNAi construct (Han et al. 2018). All the primers used to generate the over-expression constructs above were listed in Supplemental Table 1, and all of the constructs were confirmed by sequencing. The constructs were introduced into Agrobacterium strain EHA105. The wild type Kitaake was used as the recipient for Agrobacterium-mediated transformation as described previously to generate the transgenic rice. Homozygous T_3_ or T_4_ plants were taken for the following field test. The construction of the carrier and genetic transformation of the vectors were completed in the laboratory of Wan Jianmin, Institute of Crop Science, Chinese Academy of Agricultural Sciences.

The experiment was conducted at the science and technology park of Jiangxi agricultural university, Nanchang, Jiangxi province of China in 2017 (28°46′N, 115°50′E, altitude: 48.8 m, annual average temperature: 17.5 °C, average annual sunshine:1720.8 h, annual average evaporation: 1139 mm, and average annual rainfall: 1747 mm). Wild type and transgenic lines were planted in the field for measurement of physical characteristics, gene expression, gene profile, and physiological and biochemical indicators at different growth stages. Experimental plants were also established hydroponically under controlled conditions in a growth chamber for stress treatments consisting of drought, high temperature and darkness. Background conditions in the incubator were 12 h/12 h (day/night), 28 °C/25 °C (day/night), 600/0 μmol m^− 2^ s^− 1^ (day/night), and 75% relative humidity. Except as specified below, these settings remained constant. Equal numbers of seedlings at the 4–5 leaf stage were selected for each stress treatment. The drought stress treatment consisted of 4 days at 30% relative humidity with application of 20% (w/w) polyethylene glycol (PEG-6000) solution. The high temperature stress treatment consisted of 5 d at 42 °C/42 °C (day/night) using an artificial climate box thermostat. The darkness stress treatment consisted of 6 d at 0/0 mol m^− 2^ s^− 1^(day/night). Samples were collected every 24 h with acquisition of main stem and fully expanded leaves, then immediately frozen with liquid nitrogen and stored at − 80 °C. These samples were used to analyse the expression pattern of the *OsXDH* gene under different stress conditions.

To test the effect of metabolite supplementation, allantoin solutions of 0 mmol/L, 5 mmol/L, and 10 mmol/L were sprayed on rice leaves every 5 days from the beginning of the full heading stage for a total of 5 times. Allantoin-treated leaves at the top of the main stem were harvested at 5 d, 10 d, 15 d, 20 d, and 25 d. Three replicates were used for statistical evaluation of the results. All samples were frozen immediately with liquid nitrogen and stored at − 80 °C.

### Quantitative RT-PCR

Total RNA was isolated from seedling leaves using TaKaRa MiniBEST Plant RNA Extraction Kit (TaKaRa, China). About 0.5 μg total RNA was reverse-transcribed to the first-strand cDNAs using PrimeScript TM RT Master Mix (TaKaRa, China). Real-time quantitative PCR (qRT-PCR) detection was performed on a CFX96 Real-Time PCR Detection System using SYBR® Premix Ex Taq TM II (TaKaRa, China). The assaying genes included the upstream-downstream genes of *OsXDH* such as *UO* (LOC4324793), *ALN* (LOC4337428), and *AAH* (LOC4341777), and leaves senescence related genes such as *SGR* (LOC4347672), *WRKY23* ((LOC4324161), *WRKY53* (LOC4338474), *GH27* (LOC4323975), *Pse (t)* (LOC4337812), *ACD1* (LOC4331611), and *XERO1* (LOC4350453). *Ubi2* (LOC4332169) was used as the reference gene. Three plants were selected for each treatment group in each replicate experiment. During reverse transcription PCR (RT-PCR), *OsXDH* transcripts were co-amplified with *OsACTIN* (LOC4333919) mRNA as an internal control. All the primers used to gene expression analysis above were listed in Supplemental Table 2.

### XDH Enzyme Activity

Total protein was extracted from leaves and the protein content was determined by Coomassie blue staining. Non-denaturated polyacrylamide gel electrophoresis (PAGE) was then performed with a 50 μg sample. The gel was stained with hypoxanthine as the substrate and azotetrazolium as the chromogenic agent (Sagi et al. 1998). The relative activity of the XDH enzyme was calculated using grayscale analysis software Image J 2 x (Image J 2x Software, USA).

### Chlorophyll Content and Chlorophyll Fluorescence Parameters

Chlorophyll content was determined spectrophotometrically after extraction of shoots with 80% (v/v) acetone (Lichtenthaler and Wellburn 1983). Leaf sample (0.5 g) was collected from the second leaf on the stem of three plants for each treatment group in each replicate experiment.

Chlorophyll fluorescence parameters were measured by a PAM-2500 portable modulation chlorophyll fluorescence spectrometer (Walz, Effeltrich, Germany), and chlorophyll fluorescence parameters were determined for five flag leaves from each group at each growth stage of interest. After 20 min dark adaptation, actinic light was turned on. Measurements took place on sunny days between 9:00 AM and 11:30 AM. Upon stabilisation of fluorescence, the following fluorescence parameters were recorded: F_0_ (initial fluorescence), ΦPSII (actual PSII photochemical efficiency), qP (photochemical quenching coefficient), NPQ (non-photochemical quenching), ETR (photosynthetic electron transportation rate). F_v_/F_0_ (potential photochemical efficiency of PSII) and F_v_/F_m_ (maximum photochemical efficiency of PSII) were calculated.

### Phenotypic Observation

Plant height was measured at seedling, filling, and maturing stages. Five plants were selected for each group at random.

At the maturing stage, the yield traits of WT, *Ri3*, and *OE9* were investigated based on five biological replicates. The effective spikes per plant, grain number per spike, number of full grains, thousand kernel weight, spike length, grain length, grain width, and grain thickness were measured, and seed setting rate were calculated accordingly. One thousand full-filled seeds were selected randomly and weighed to get the thousand kernel weight.

### Allantoin and Allantoate Content

Allantoin and allantoate were extracted from leaves with 80% ethanol. Allantoin and allantoate levels were determined according to the method described in Sagi et al. (1998). Leaf sample (0.5 g) was collected from the second leaf on the stem of three plants for each treatment group in each replicate experiment.

### Antioxidant Capacity

Nitroblue tetrazolium O_2_^−^ staining of leaves was performed according to the method described by Brychkova et al. (2008). H_2_O_2_ content was determined as described by Lin and Kao (2001). MDA content was determined by thiobarbituric acid colorimetry (Chen et al. 2011).

### Statistical Analysis

Individual means and standard errors of the mean were calculated using the data from independent samples in Microsoft Excel 2007 (Microsoft, USA). IBM SPSS Statistics 22 (SPSS 22, SPSS Inc., USA) was used for statistical analysis, and the least significant difference method (LSD) was used for the inter-processing significance test. Significance was specified as *P* < 0.05 and indicated by different letters.

## Results

### Expression Pattern Analysis for *OsXDH*

**Fig. 1:**
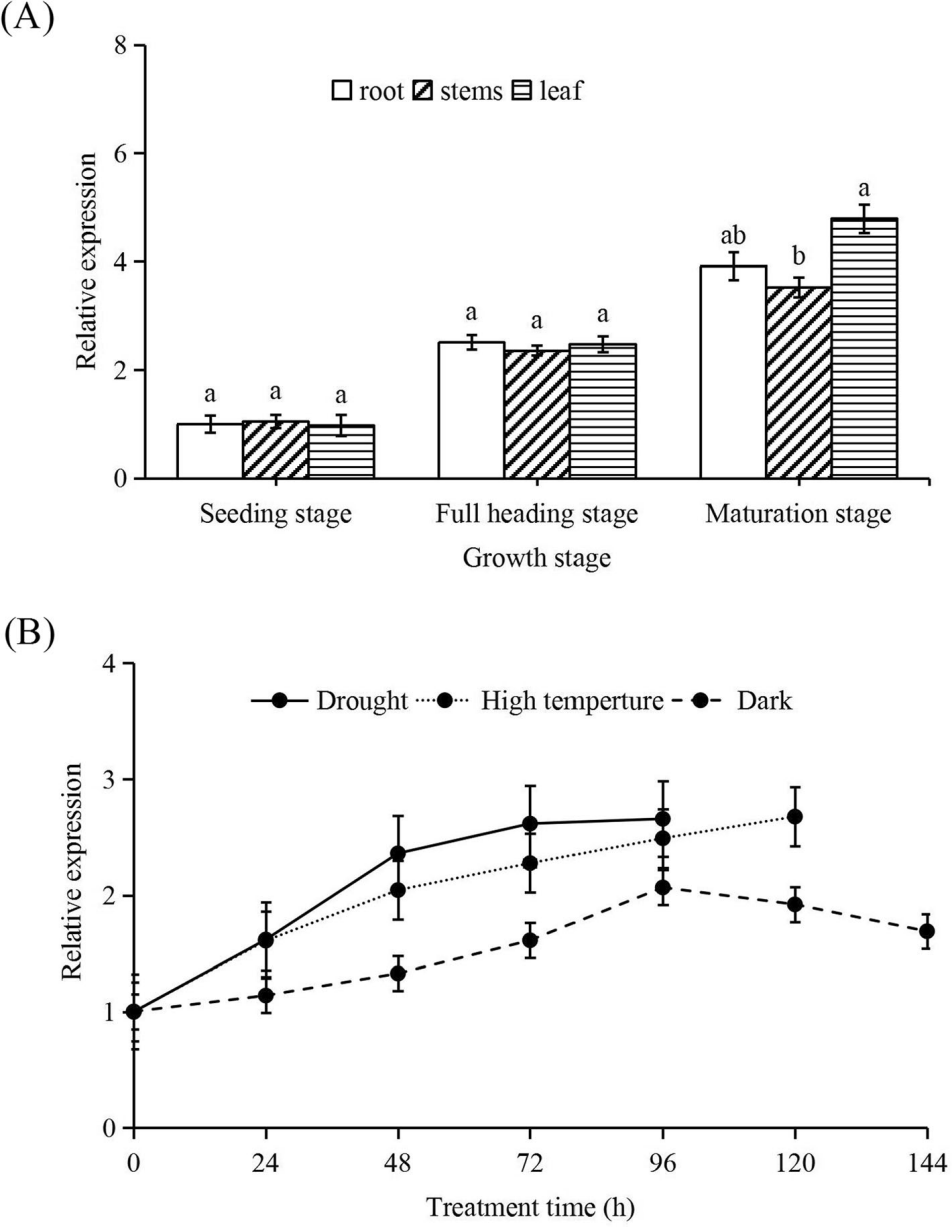
Expression pattern analysis for *OsXDH.***a** Expression level of *OsXDH* in various tissues of rice at different growth stages. The sampling at seedling stage was 14 days after sowing, and the sampling at full spike and maturity stages were 60 days and 90 days after sowing, respectively. The sampled leaves were the penultimate leaves, the sampled stems were located near the penultimate leaf, and the young roots were collected for expression pattern analysis of *OsXDH*. **b** Expression levels of *OsXDH* in rice leaves under different stresses. Equal numbers of seedlings at the 4–5 leaf stage were selected for each stress treatment, Samples were collected every 24 h with acquisition of main stem and fully expanded leaves in different adversity handling processes. Values are means±SEM, *n* = 3. Significant differences (*P* < 0.05) are denoted by different lowercase letters

In order to determine the response of *OsXDH* gene expression to ageing and stress factors, qPCR was used to analyse the *OsXDH* gene expression level in leaves, roots, stems and leaves of rice at different growth stages under different stress conditions. The results showed that there was no significant difference in the expression of *OsXDH* gene between roots, stems and leaves at the seedling and full heading stages. Expression of *OsXDH* increased at the full heading and maturation stages. Expression levels differed between roots, stems and leave at the maturation stage, with higher levels in leaves (Fig. 1a). Continuously increasing levels were seen under drought, high temperature, and dark stress conditions (Fig. 1b).

### Plant Height and Chlorophyll Content at Different Growth Stages

**Fig. 2:**
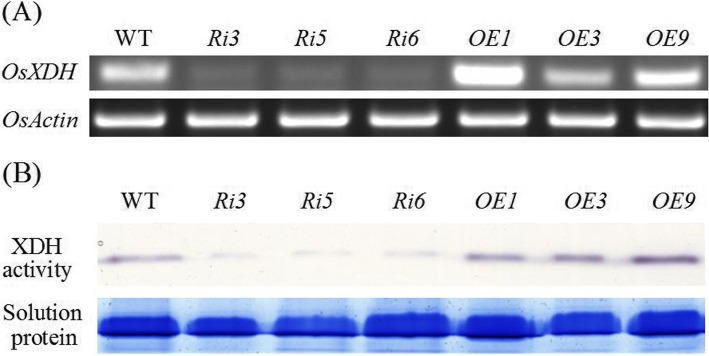
The relative expression level of *OsXDH* and XDH activity in wild-type and transgenic plants. **a** Expression analysis of *OsXDH* in rice seedlings cultured for 30 days*. Ri3*, *Ri5,* and *Ri6* were XDH interference transgenic lines, and *OE1, OE3,* and *OE9* were XDH over-expression transgenic lines*.* The internal reference gene was *OsActin*. **b** Enzyme activity analysis of XDH in rice seedlings cultured for 30 days. Soluble protein was extracted from rice seedling leaves grown for 30 days. Each lane in the gel was loaded with equal content soluble protein. XDH activity was detected in gel with hypoxanthine as substrate

**Fig. 3:**
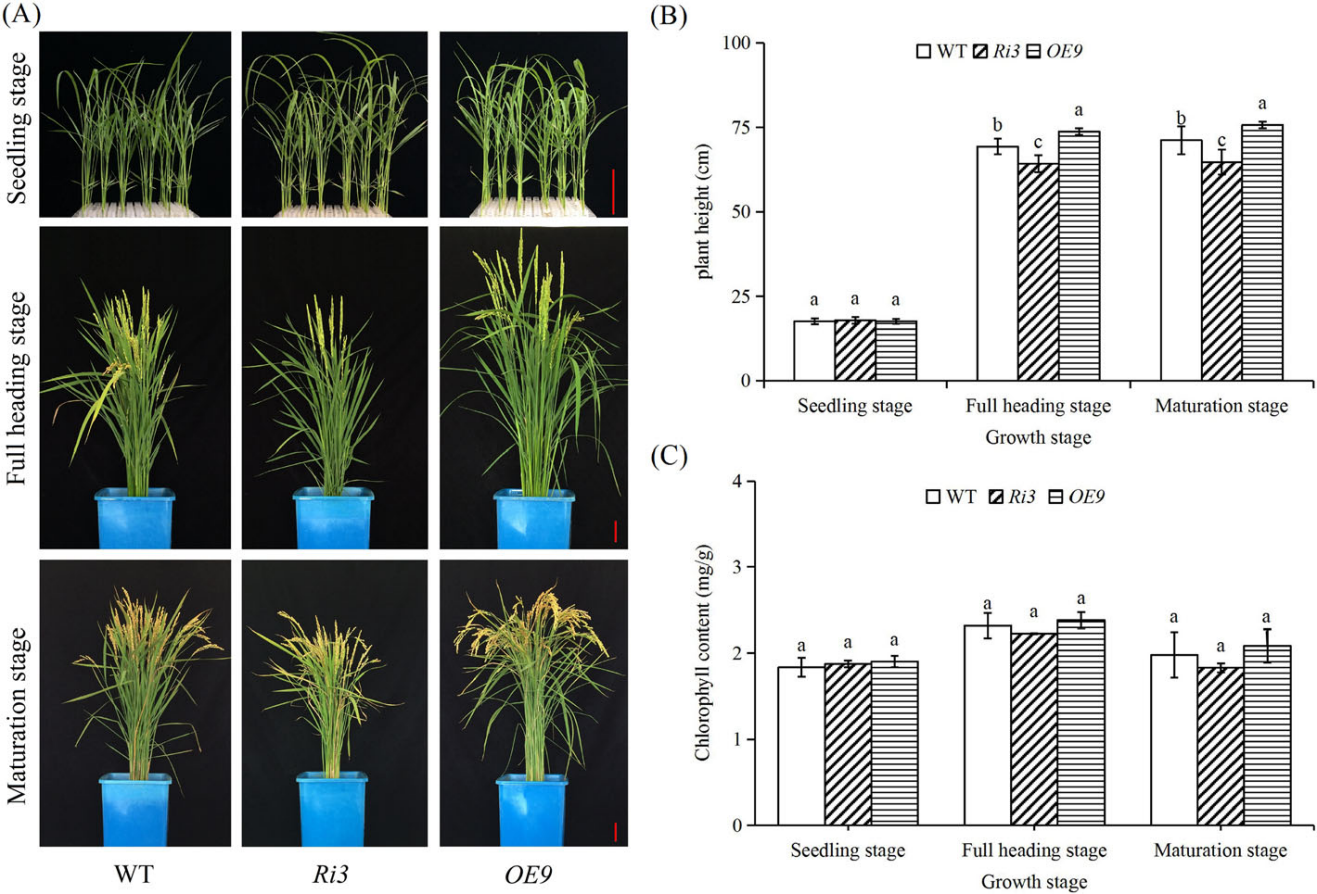
Plant height and chlorophyll content differences between wild-type and transgenic plants at different growth stages. **a** Appearance, **b** plant height, and **c** chlorophyll content of WT, *Ri3* and *OE9* at different growth stages. The seedling, full heading and maturation stages of rice were grown to 14d, 60d, and 90d after sowing, respectively. Values are means±SEM, *n* = 5(**b**) and 3(**c**). Significant differences (*P* < 0.05) are denoted by different lowercase letters

XDH interference (*Ri3*, *Ri5, and Ri6*) and over-expression (*OE1*, *OE3,* and *OE9*) transgenic lines were constructed for explore the function of *OsXDH*. We measured *OsXDH* gene expression and XDH enzyme activity to identify the transgenic lines with significantly decreased and increased XDH activity (Fig. 2). *Ri3* and *OE9* were used for the next experiment. Plant height and chlorophyll content of the wild-type and transgenic plants were measured at seedling, full heading and maturation stages. At the seedling stage, no phenotypic difference between lines was seen. At later stages, plant height and chlorophyll content were lower in the interference line, and higher in the over-expression line, than in wild-type plants (Fig. 3). This indicates that the influence of *OsXDH* gene expression on rice under normal conditions requires long-term accumulation.

### Chlorophyll Fluorescence Parameters at Different Stages

To study the dynamics of XDH activity on rice growth habit and physiology, as well as the effects on photosynthesis, we measured chlorophyll fluorescence parameters (F_v_/F_m_, ΦPSII, qP, NPQ, ETR, F_v_/F_0_) of the transgenic lines at seedling, tillering, full heading, filling and maturation stages. F_v_/F_m_ represents the maximum photosynthetic efficiency of photosystem II and reflects the potential maximum photoenergy conversion efficiency of the plants. ΦPSII is the actual photosynthetic efficiency of photosystem II, indicating the real light conversion efficiency of the photosynthetic mechanism at the time of measurement. Fluorescence quenching caused by photosynthesis is called photochemical quenching (qP), and fluorescence quenching caused by heat dissipation is called non-photochemical quenching (NPQ). The plant’s photosynthetic activity level is represented by qP, and its ability to dissipate excess light energy to heat (light protection) is shown by NPQ. ETR is the photosynthetic electron transfer rate, and F_v_/F_0_ reflects the potential activity of photosystem II (PSII). Our results showed that all chlorophyll fluorescence parameters first rose and then fell as the plants grew, reaching maximum values at full heading stage (Table 1). Thereafter, differences between plant lines began to appear. This was consistent with the lines’ chlorophyll levels (Fig. 1), indicating that XDH begins to influence rice phenotype and physiology at the full heading stage, or during tillering prior to full heading. Once the stage is reached, the chlorophyll fluorescence parameters for the interference line are lower, and for the over-expression line higher, than those for the wild type. These results showed that more photosystem reaction centres are open in *OE9* plants in the late growth period, which could thus maintain high photochemical activity alongside a strong self-protection mechanism. In *Ri3* plants, PSII actual light energy conversion efficiency is lower, potential active centres are damaged, and the reactions of photosynthesis are restrained, ultimately resulting in a decline in photosynthetic rate.

**Table 1:** Chlorophyll fluorescence kinetic parameters in wild-type and transgenic lines at different growth stages

parameters	Lines	Seedling stage	Tillering stage	Full heading stage	Filling stage	Maturation stage
Fv/Fm	WT	0.748 ± 0.016 a	0.806 ± 0.005 a	0.823 ± 0.016 a	0.804 ± 0.021 ab	0.731 ± 0.023 a
	*Ri3*	0.755 ± 0.007 a	0.801 ± 0.003 a	0.785 ± 0.018 b	0.751 ± 0.063 b	0.685 ± 0.007 b
	*OE9*	0.752 ± 0.004 a	0.812 ± 0.017 a	0.841 ± 0.013 a	0.838 ± 0.025 a	0.732 ± 0.021 a
ΦPSII	WT	0.185 ± 0.019 a	0.221 ± 0.012 a	0.275 ± 0.012 a	0.248 ± 0.031 a	0.178 ± 0.014 a
	*Ri3*	0.198 ± 0.019 a	0.223 ± 0.011 a	0.235 ± 0.012 b	0.165 ± 0.011 b	0.141 ± 0.009 b
	*OE9*	0.203 ± 0.022 a	0.228 ± 0.009 a	0.291 ± 0.013 a	0.257 ± 0.014 a	0.196 ± 0.012 a
qP	WT	0.241 ± 0.025 a	0.294 ± 0.007 a	0.331 ± 0.023 b	0.304 ± 0.019 b	0.222 ± 0.008 a
	*Ri3*	0.279 ± 0.041 a	0.311 ± 0.024 a	0.296 ± 0.013 b	0.274 ± 0.021 b	0.141 ± 0.024 b
	*OE9*	0.273 ± 0.028 a	0.317 ± 0.015 a	0.368 ± 0.017 a	0.355 ± 0.021 a	0.247 ± 0.027 a
NPQ	WT	0.112 ± 0.013 a	0.132 ± 0.006 a	0.153 ± 0.007 a	0.135 ± 0.012 a	0.107 ± 0.023 a
	*Ri3*	0.099 ± 0.023 a	0.136 ± 0.009 a	0.136 ± 0.013 a	0.111 ± 0.025 a	0.069 ± 0.004 b
	*OE9*	0.129 ± 0.026 a	0.145 ± 0.014 a	0.166 ± 0.025 a	0.154 ± 0.051 a	0.110 ± 0.014 a
ETR	WT	27.741 ± 3.159 a	33.503 ± 0.467 a	38.930 ± 4.319 ab	36.780 ± 1.631 ab	24.460 ± 3.051 ab
	*Ri3*	32.251 ± 4.236 a	32.663 ± 3.695 a	34.060 ± 2.857 b	31.497 ± 4.367 b	20.643 ± 0.771 b
	*OE9*	31.447 ± 3.374 a	34.820 ± 1.146 a	43.217 ± 4.128 a	41.187 ± 2.093 a	28.913 ± 3.019 a
Fv/Fo	WT	2.975 ± 0.236 a	4.036 ± 0.223 a	5.059 ± 0.184 a	4.889 ± 0.246 ab	2.275 ± 0.147 a
	*Ri3*	2.973 ± 0.072 a	4.012 ± 0.227 a	4.142 ± 0.339 b	4.416 ± 0.495 b	1.951 ± 0.237 a
	*OE9*	2.764 ± 0.251 a	4.107 ± 0.145 a	5.547 ± 0.269 a	5.346 ± 0.346 a	2.326 ± 0.224 a

The seedling, tillering, full heading, filling, and maturation stages were grown to 14d, 35d, 60d, 80d, and 90d after sowing, respectively. The penultimate leaves were selected at seedling and tillering stages, and sword leaves are selected for the determination of chlorophyll fluorescence kinetic parameters at full heading, filling, and maturation stages. *Fv/Fm* the maximum photosynthetic efficiency of photosystem II; *ΦPSII* Actual photosynthetic efficiency of photosystem II, *qP* Photochemical quenching, *NPQ* Non-photochemical quenching, *ETR* Photosynthetic electron transfer rate, *Fv/Fo* Potential activity of photosystem II. Values are means±SEM, *n* = 5. Significant differences (*P* < 0.05) are denoted by different lowercase letters

### Yield Traits of Transgenic Lines

The changes in XDH activity in transgenic plants affected the chlorophyll content and photosynthetic efficiency of rice leaves, which were closely related to dry matter accumulation and yield. We measured the yield traits spike shape, effective spikes per plant, seed-setting rate, grain number per ear, 1000-grain weight, and grain size of wild-type and transgenic plants (Table 2, Supplementary Fig. S1). The effects of XDH on yield traits were mainly focused on the seed-setting rate and 1000-grain weight. Setting rate in *OE9* plants was higher than in wild-type plants, and significantly higher than in *Ri3* plants. The 1000-grain weight for *OE9* plants was significantly higher for *Ri3* plants, although there was no significant difference between *OE9* and wild-type. Given the difference in 1000-grain weight between the three lines, we then performed statistical analyses of grain length, grain width and grain thickness, we found that grain length and grain width of the interference line were lower than that of the wild-type, and the over-expression line were higher than that of wild-type.

**Table 2:** Yield component differences between wild-type and transgenic lines

Lines	Effective spikes per plant	Grain number per spike	Setting rate (%)	Thousand kernel weight (g)	Spike length (cm)	Grain length (mm)	Grain width (mm)	Grain thickness (mm)
WT	35.33 ± 3.21 a	35.30 ± 5.04 a	74.97 ± 2.64 a	24.15 ± 1.11 ab	11.36 ± 0.22 b	7.43 ± 0.28 a	3.30 ± 0.11 ab	2.30 ± 0.20 a
*Ri3*	32.00 ± 4.00 a	41.44 ± 8.61 a	63.03 ± 4.87 b	22.51 ± 1.76 b	10.97 ± 0.47 b	7.01 ± 0.19 b	3.24 ± 0.05 b	2.17 ± 0.04 a
*OE9*	36.67 ± 3.21 a	42.39 ± 6.12 a	79.63 ± 3.01 a	24.88 ± 0.53 a	12.75 ± 0.19 a	7.52 ± 0.22 a	3.34 ± 0.08 a	2.25 ± 0.07 a

All grains measured after harvest and drying for 3 days. Values are means±SEM, *n* = 5. Significant differences (*P* < 0.05) are denoted by different lowercase letters

### Antioxidant Capacity

**Fig. 4:**
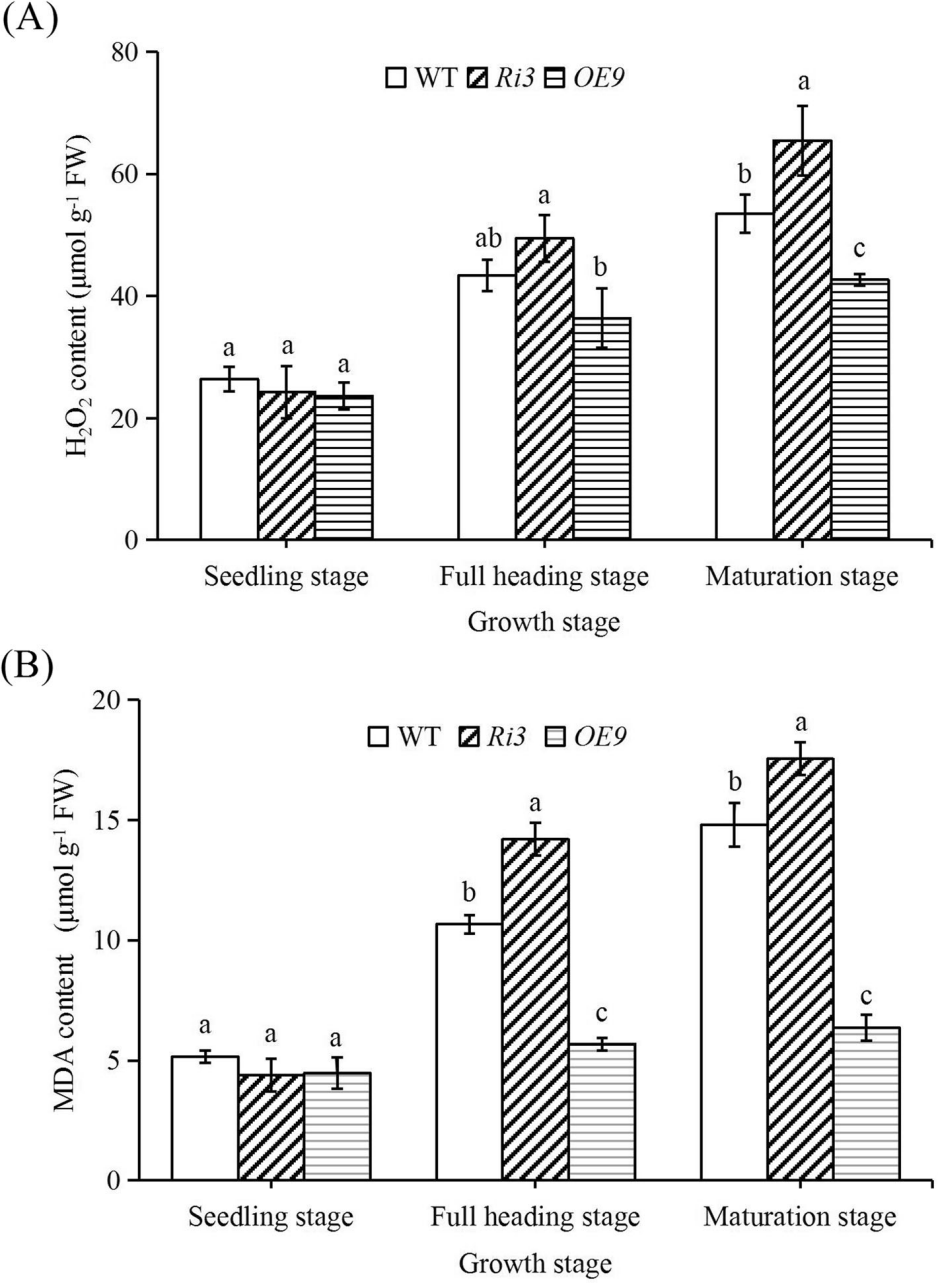
Differences in reactive oxygen metabolism in wild-type and transgenic lines at different growth stages. **a** H_2_O_2_ content. **b** MDA content. The seedling, full heading and maturation stages of rice were grown to 14d, 60d, and 90d after sowing, respectively. The penultimate leaves were selected at seedling stage, and sword leaves are selected for the determination of H_2_O_2_ and MDA content at full heading and maturation stages. FW, fresh weight. Values are means±SEM, *n* = 5. Significant differences (*P* < 0.05) are denoted by different lowercase letters

The biological free radical injury hypothesis holds that the ageing process is the accumulation of maladjustments in reactive oxygen metabolism. O_2_^−^ (Supplementary Fig. S2), H_2_O_2_ and MDA (Fig. 4) content were measured in transgenic and wild-type plants to determine the ROS level and degree of membrane lipid peroxidation at different growth stages. Levels of O_2_^−^, H_2_O_2_ and MDA in all plants were lower at the seedling stage, with no significant difference between lines. O_2_^−^, H_2_O_2_ and MDA continued to increase through full heading and maturation stages, with ROS content and membrane lipid peroxidation significantly higher in *Ri3* plants than in wild-type and *OE9* plants, and *OE9* contains the lowest amount of them.

### XDH Activity, Allantoin and Allantoate Content

**Fig. 5:**
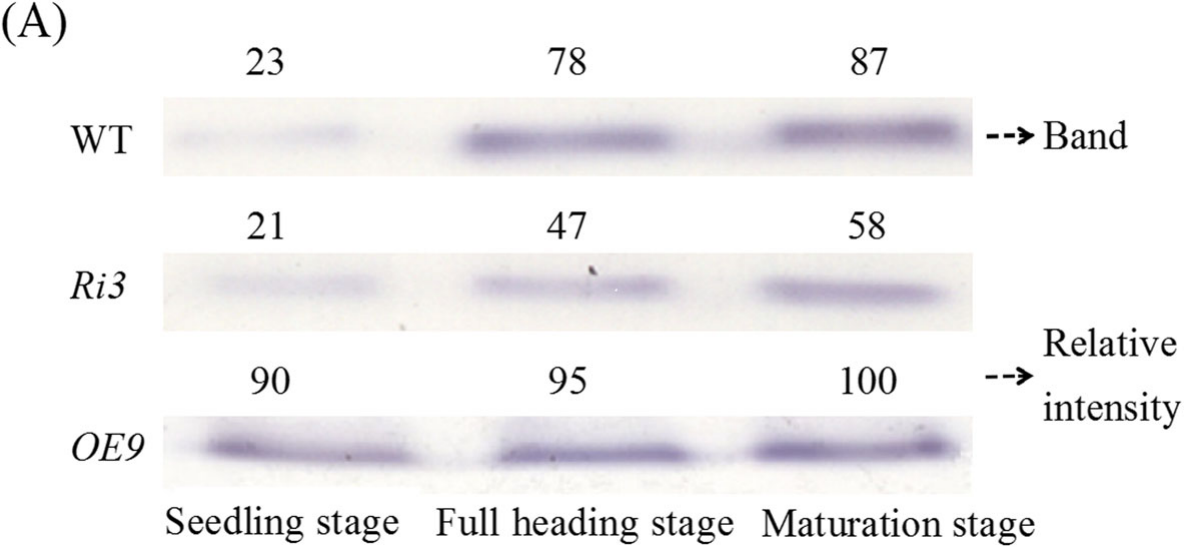
XDH enzyme activity in wild-type and transgenic lines at different growth stages. The seedling, full heading and maturation stages of rice were grown to 14d, 60d, and 90d after sowing, respectively. The penultimate leaves were selected at seedling stage and sword leaves of transgenic plants were selected for extraction soluble protein at full heading and maturation stages. Each lane in the gel was loaded with equal content soluble protein. XDH activity was detected in gel with hypoxanthine as substrate. Numbers above the lanes indicate relative intensity obtained by scanning the formazan bands with a computing laser densitometer using Image J 2x software

**Fig. 6:**
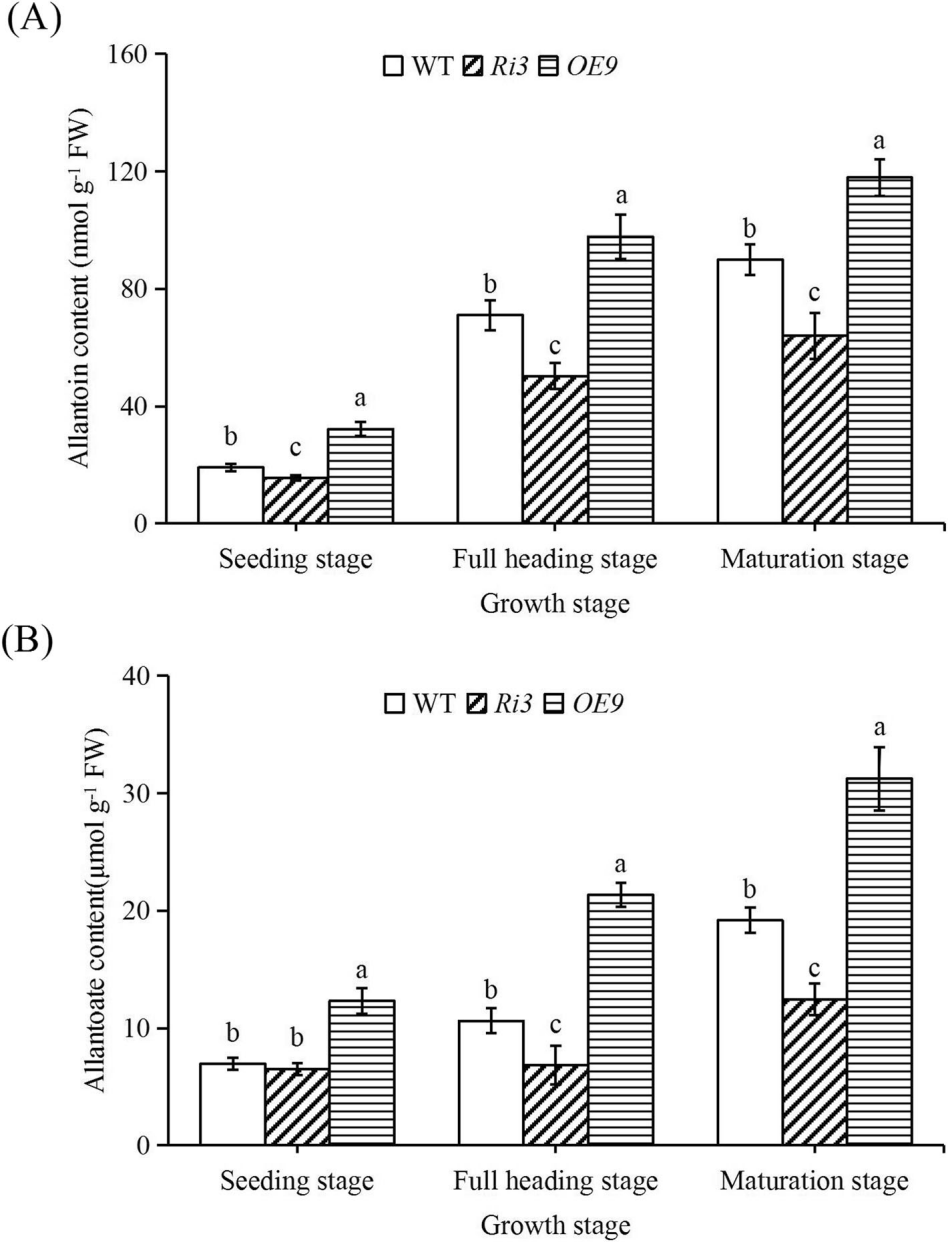
Allantoin and allantoate content in wild-type and transgenic lines at different growth stages. The seedling, full heading and maturation stages of rice were grown to 14d, 60d, and 90d after sowing, respectively. The penultimate leaves were selected at seedling stage, and sword leaves are selected for the determination of allantoin (**a**) and allantoate (**b**) content at full heading and maturation stages. FW, fresh weight. Values are means±SEM, *n* = 3. Significant differences (*P* < 0.05) are denoted by different lowercase letters

Changes in *OsXDH* gene expression levels were accompanied by changes in XDH activity and purine metabolism at the seedling, full heading and maturation stages (Figs. 5 and 6). The XDH activity of all lines was significantly increased at the full heading and maturation stages, the XDH activity in the over-expression line was higher than wild-type, and the interference line were lower than that of wild-type throughout the growth period. The content of allantoin and allantoate in all lines showed the same trend as the XDH enzyme activity.

### Expression Levels of Genes Upstream and Downstream of OsXDH and Senescence Related Genes

**Fig. 7:**
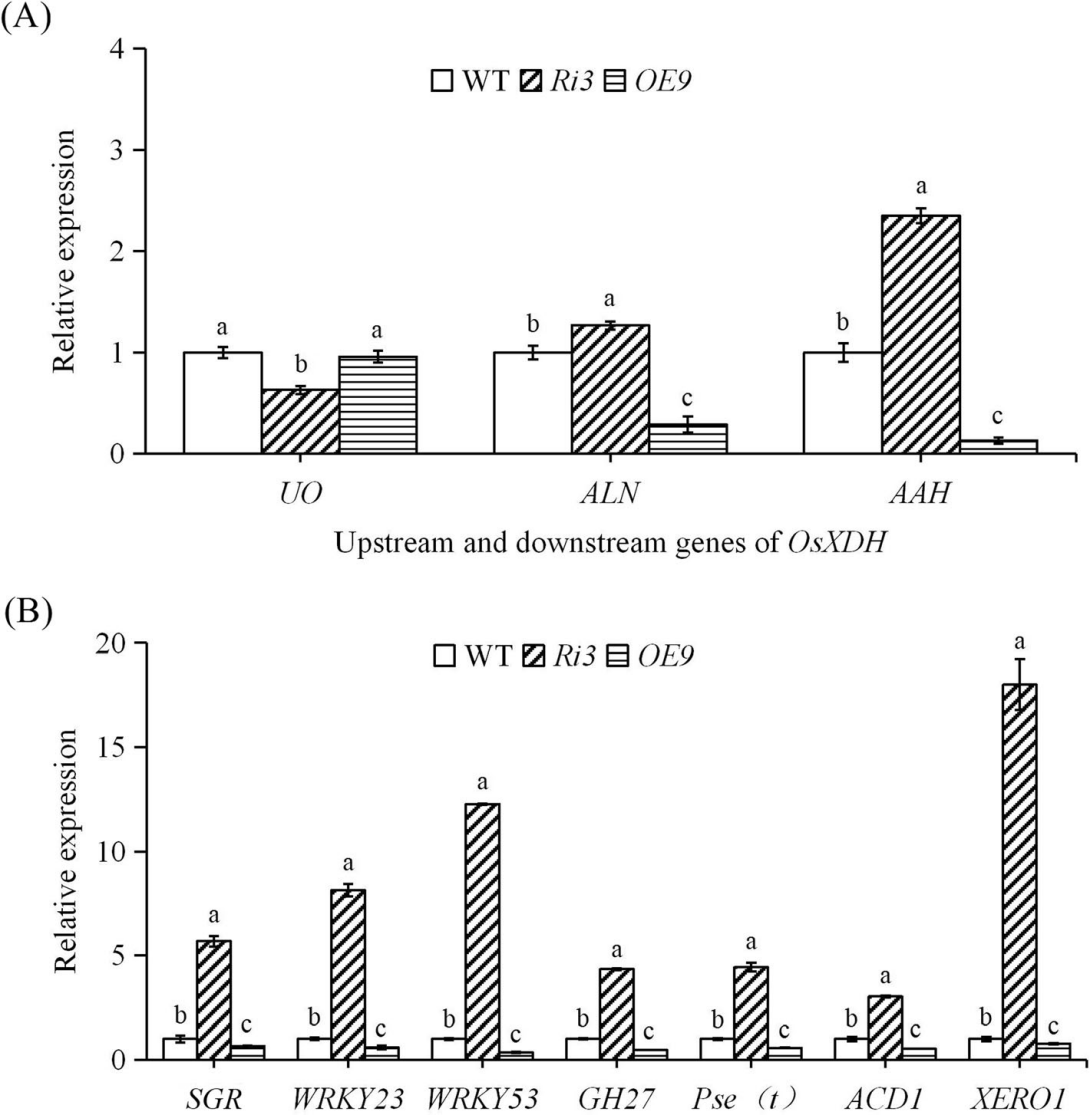
Expression analysis of genes upstream and downstream of *OsXDH* and of senescence related genes at the maturation stage. The maturation stage of rice were grown to 90d after sowing. Sword leaves are selected for the determination of expression of upstream-downstream genes of *OsXDH* (**a**) and leaves senescence related genes (**b**) at maturation stage. *Ubi2* served as an internal control. Values are means±SEM, *n* = 3. Significant differences (*P* < 0.05) are denoted by different lowercase letters

Studies have shown that XDH is involved in regulating the ageing process of rice leaves (Brychkova et al. 2008). We analysed expression levels of genes upstream-downstream of *OsXDH* and of senescence related genes in rice leaves during the filling stage. This enabled identification of the mechanism by which XDH regulates the senescence of rice leaves (Fig.7). The results showed that the urate oxidase gene (*UO*) expression levels in the interference line were significantly lower than those in the over-expression line and wild-type lines. The allantoinase (*ALN*) and allantoate amidinohydrolase (*AAH*) genes had significantly higher expression in the *Ri3* plants than the in *OE9* or wild-type plants, with *OE9* plants showing the lowest levels. The senescence-related genes *SGR*, *WRKY23*, *WRKY53*, *GH27*, *Pse(t)*, *ACD1*, and *XERO1* in *Ri3* plants had the highest expression levels, followed by those in the wild-type plants, with *OE9* plants showing the lowest levels.

### Exogenous Allantoin Alleviates Chlorophyll Degradation and Enhances Antioxidant Capacity

**Fig. 8:**
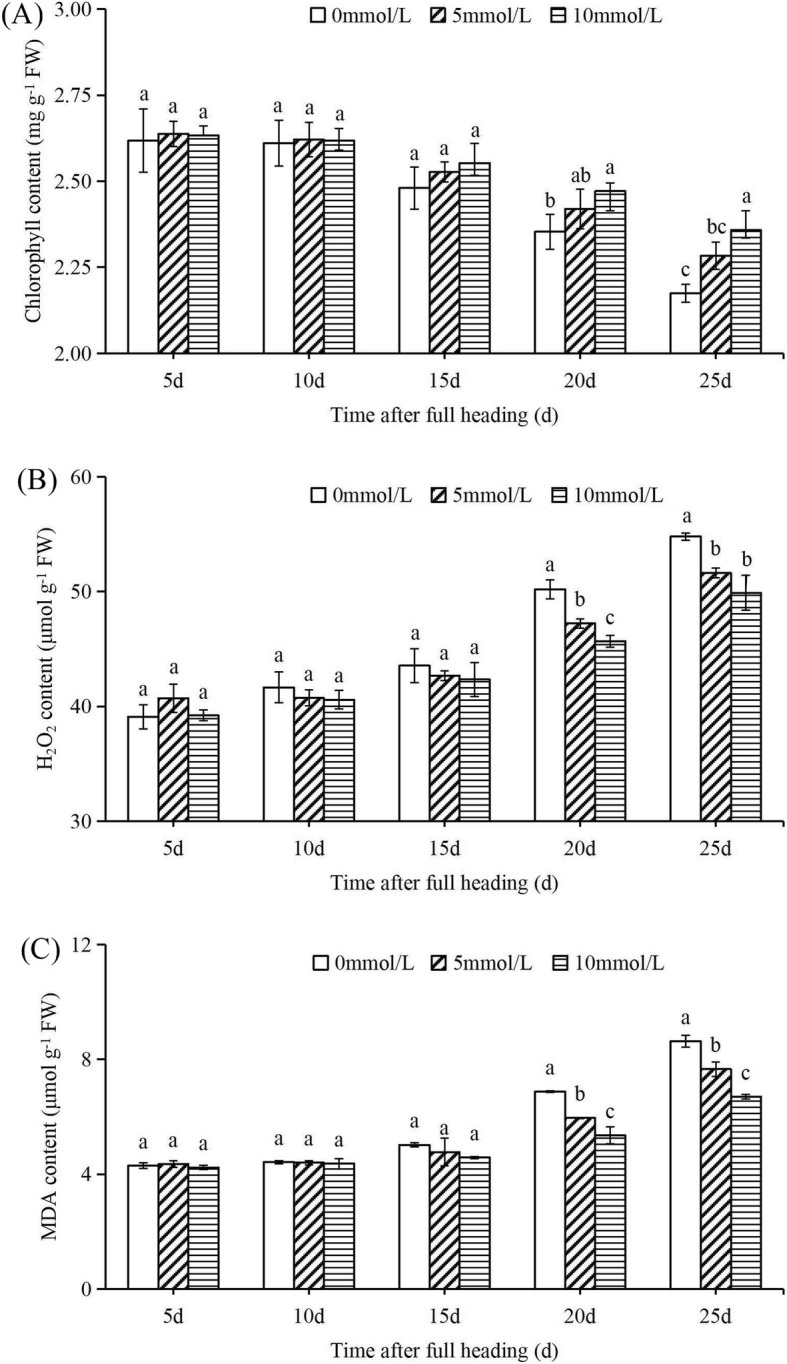
Chlorophyll, H_2_O_2,_ and MDA content of rice after exogenous spraying of allantoin at different concentrations after full heading. After 60 days of growth, the rice reached the peak stage, and then the leaf was sprayed with different concentrations of allantoin (0 mmol/L, 5 mmol/L, and 10 mmol/L) every 5 days. The sword leaves of rice were used for the determination of chlorophyll (**a**), H_2_O_2_ (**b**) and MDA (**c**) content. FW, fresh weight. Values are means±SEM, *n* = 3. Significant differences (*P* < 0.05) are denoted by different lowercase letters

To determine whether purine metabolites actually contribute to delay rice leaf senescence, we measured chlorophyll content by spraying exogenous allantoin on rice leaves after the full heading stage (Fig. 8). During this extended growth period, chlorophyll content in the leaves decreased gradually, especially after 15 days following full heading. The higher the concentration of exogenous allantoin, the slower the rate of decrease of chlorophyll content. At 25 d following the full heading stage, the chlorophyll content of rice leaves sprayed with 10 mmol/L exogenous allantoin was 0.183 mg/g more than the control leaves sprayed with 0 mmol/L.

We also measured the H_2_O_2_ and MDA content in the leaves sprayed with exogenous allantoin (Fig. 8b, c). During this extended growth period, the content of H_2_O_2_ and MDA increased gradually, especially after 15 days following full heading. The higher the concentration of exogenous allantoin, the slower the accumulation of H_2_O_2_ and MDA. The H_2_O_2_ and MDA content of leaves sprayed with 10 mmol/L exogenous allantoin was 4.905 μmol/g and 1.937 μmol/g, respectively, less than controls sprayed with 0 mmol/L at 25 d following full heading stage.

## Discussion

### XDH Is Involved in the Regulation of Rice Leaf Senescence

Preliminary analysis of the *OsXDH* expression profile in rice showed that expression levels increased with the onset of the reproductive growth phase, and in the presence of adverse environmental factors. Dark stress and senescence can induce and regulate purine metabolism, which is in turn regulated by its own feedback; this cycle is involved in dark stress and leaf senescence tolerance in Arabidopsis (Brychkova et al. 2008). Insertion and over-expression of the grape gene *VvXDH* in *Arabidopsis thaliana* improved its tolerance to salt stress (You et al. 2017). High concentrations of NO_3_^−^ and NH_4_^+^ ions can induce the expression of *ZmXDH* in maize and improve the activity of XDH (Katalin et al. 2000). In previous studies, we found that the homology of XDH was relatively high among different advanced plants (Han et al. 2017). The regulation mechanism of XDH involved in plant leaf premature senility involves multiple processes such as metabolism of nitrogen, reactive oxygen species, and hormones in Arabidopsis, corn, pea and other advanced plants. We speculated that XDH in rice was involved in regulating the normal ageing process, in addition to being induced by abiotic stress (Brychkova et al. 2008; Katalin et al. 2000; You et al. 2017; Zdunek-Zastocka and Lips 2003).

Senescence is the final stage of the process of plant growth and development. It is an orderly self-disintegration process regulated by genetic factors. The first organ to decay is the leaf, in which chloroplasts disintegrate, photosynthesis weakens, and nucleic acid, protein and lipid levels fall. Leaf senescence is not only regulated by endogenous signal molecules such as plant hormones, but also affected by environmental factors such as drought, salinity, temperature stress and pathogen infection (Rogers 2017). In the later stages of crop reproductive growth and development, leaf senescence leads to a decrease in photosynthetic efficiency, which affects the accumulation of sugars during seed formation and ultimately affects crop yields (Guiboileau et al. 2010). *Ri3* and *OE9* were transgenic lines with *OsXDH* gene interference and over-expression, respectively. Compared with the wild type, the phenotypes of both lines were normal at the seedling and tillering stages; differences appeared at the full heading stage. These manifested as reduced plant height, chlorophyll content, and chlorophyll fluorescence kinetic parameters in the interference line. The lowered chlorophyll content led to a reduced photosynthetic rate; the decrease in photosynthetic products led to lowered seed setting rate and 1000-grain weight. (Fig.3, Tables 1 and 2). By contrast, the over-expression line showed strong growth advantages. Compared with the wild type, it had improved plant height, chlorophyll content, chlorophyll fluorescence kinetic parameters, seed setting rate and 1000-grain weight. Together, these results indicated that XDH was involved in the regulation of rice leaf senescence and ultimately affected the rice yields. Similar results have been obtained in studies of *Arabidopsis thaliana*. Interference in *AtXDH* gene expression in *Arabidopsis thaliana* can hinder plant growth, accelerate leaf cell death, and significantly reduce chlorophyll content (Han et al. 2018).

### XDH Is Involved in the Regulation of Reactive Oxygen Metabolism in Rice Leaves

We found that the levels of superoxide ions (O_2_^−^), H_2_O_2_ and MDA in *OsXDH* interference lines were significantly higher than in the wild-type at the full heading and maturation stages, while levels in the over-expression line were lower. Previous studies have shown that damage to rice seedlings caused by dark stress could be alleviated by applying allantoin on the leaf surface (Brychkova et al. 2008). Exogenous allantoin, allantoate and uric acid can reduce the content of H_2_O_2_ and O_2_^−^ in the leaves of *Arabidopsis thaliana*, increase chlorophyll content, and reduce the damage caused by adverse environmental factors (Brychkova et al. 2008; Irani and Todd 2018; Watanabe et al. 2010; Watanabe et al. 2014). Following measurement of purine metabolite content in our study, we found that levels of allantoin and allantoate in the over-expression line were significantly higher than those in the wild type at the full heading and maturation stages, whereas these levels in the interference line were significantly lower than in the wild-type. Spraying exogenous allantoin on rice leaves every 5 days from the beginning of the full heading stage was successful in alleviating chlorophyll degradation and reducing H_2_O_2_ and MDA content in rice leaves. We also analysed the expression levels of key enzymes in purine metabolism. Compared with the wild-type, there was no significant change in the expression level of *UO* and the expression levels of *ALH* and *AAH* genes decreased in over-expression line, thus promoting the accumulation of allantoin. Studies have shown that XDH was involved in the regulation of reactive oxygen metabolism in rice leaves.

### Changes of XDH Activity Affect the Expression of Ageing Related Genes

*ACD1* (accelerated cell-death 1) is an accelerated death gene in plants, which is involved in the regulation of pheophorbide a oxygenase activity. The over-expression of *ACD1* leads to the degradation of chlorophyll a, accelerating plant senescence and death (Tanaka et al. 2003). The Arabidopsis WRKY protein is a family of plant-specific zinc finger transcription factors involved in the regulation of pathogen defence, injury response, hair follicle development, and gene expression during ageing. *WRKY53* plays a role in the complex transcription factor signal network regulating senescence-specific gene expression, and its Arabidopsis over-expression lines show premature ageing (Ay et al. 2009; Miao et al. 2004). *OsWRKY23* is a novel factor regulating pathogen response and dark leaf senescence. Over-expression of *OsWRKY23* can promote leaf senescence in dark environments (Jing et al. 2009). In senescent leaves, *SGR* induces the disintegration of light-harvesting chlorophyll binding protein (LHCPII) by direct interaction as part of programmed chlorophyll degradation, and *SGR* over-expression transgenic rice produced yellow-brown leaves (Park et al. 2007). *XERO1* is a dehydrin gene, which is involved in resisting adversity and delaying the ageing process (Brychkova et al. 2007). *GH27* is involved in a variety of signal transduction processes in plant growth, development, and response to the environment; it thus plays an important role in plant activity (Barleben et al. 2005; Knoch et al. 2014). A premature senescence gene *Pse(t)* was located in the T-DNA insertion mutant in of ‘Zhonghua 11’, and the mutation of this gene could lead to most leaves became yellow and wilted as senescence progressed at milk stage (Li et al. 2005). In the present study, these genes were activated in the later stages of leaf senescence, stress, and injury, and the increase of gene expression accelerated plant senescence. The expression of these genes in the interference line was substantially increased, and highly significant in comparison to the wild-type and over-expression lines, the latter showing the lowest levels of expression. In summary, compared with the wild-type, the ageing process of the interference line was accelerated, while that of the over-expression line was delayed. XDH activity is thus involved in the regulation of ageing-related gene expression, and indirectly regulates the ageing process of rice.

Plants enhance their adaptability to the environment by accelerating the recycling of internal resources when under stress from senescence and adversity (Lim et al. 2003). Within this process, XDH catalyses the production of intermediate products in purine metabolism. Ureides, including allantoin and allantoate, play an important role in the recycling and reuse of nitrogen (Brychkova et al. 2008). Ureides are composed of 4 C and 4 N atoms, a high N/C ratio compared with, for example, glutamine (Gln, 0.4) and aspartame (Asn, 0.5). The high N/C ratio leads to easy transportation within plants (Zrenner et al. 2006). This low-energy N/C ratio compound plays an important role in plant survival under stress (Omarov et al. 1998). It was found that a large amount of uric acid accumulated during plant senescence and seed germination, and nitrogen fixed in soybean root nodules was mainly transported elsewhere in the plants in the form of ureides (Fujihara and Yamaguchi 1978). Large amounts of allantoin and allantoate accumulated in the over-expression line in the present study, which promoted the transport and accumulation of nitrogen. We speculate that this is another major factor in the formation of high-yield traits. Currently, there is a lack of in-depth research on the regulation of nitrogen metabolism by XDH, although we intend to make this a future research direction for our laboratory.

## Conclusion

The primary result reported by this paper is the mechanism by which XDH activity enhancement delays rice leaf senescence. The outcomes of the study indicate that the increase of XDH activity can enhance the antioxidant capacity of rice and delay the aging of rice leaves. Allantoin, a purine metabolite, play important role in cascade of events responsible for leaf blade premature aging regulating the rice antioxidants induction or reduction. The antioxidant capacity of leaves is closely related to chlorophyll synthesis and degradation as well as photosynthetic capacity, which ultimately affects the accumulation of photosynthetic products. In summary, XDH can delay the senescence of rice leaves, improve the accumulation of photosynthetic products and increase the grain yield of rice.

## Supplementary information

**Additional file 1: Table S1.** Primers of vector construction. **Table S2.** Primers of gene expression analysis. **Figure S1.** Comparison of panicle length and grain type in wild-type and transgenic lines. (A) Spike shape. 6 (B–D) Grain shape. The white band represents a length of 1cm. **Figure S2.** Superoxide ion (O_2_^−^) levels in wild-type and transgenic lines at different growth stages. Leaves were stained with nitroblue tetrazolium (NBT) for 2 h as described in the Materials and Methods section. The black band represents a length of 100 μm.

## Data Availability

The datasets used and/or analysed during the current study are available from the corresponding author on reasonable request.

## Abbreviations

*AAH*: Allantoate amidinohydrolase
*ACD1*: Accelerated cell-death 1
*ALN*: Allantoinase
F_v_/F_0_: Potential activity of photosystem II
F_v_/F_m_: Maximum photosynthetic efficiency of photosystem II
*GH27*: Glycoside hydrolase 27
MDA: Malonaldehyde
NPQ: Non-photochemical quenching
*OE9*: An *OsXDH* over-expression transgenic line
PAGE: Polyacrylamide gel electrophoresis
*Pse (t)*: Premature senescence, tentatively
qP: Photochemical quenching
*Ri3*: An *OsXDH* RNA interference line
RT-PCR: Reverse Transcription PCR
*SGR*: Staygreen
*UO*: Urate oxidase
WT: Wild type
XDH: Xanthine dehydrogenase
*XERO1*: Dehydrin
ΦPSII: Actual photosynthetic efficiency of photosystem II

## References

[CR1] Ay N, Irmler K, Fischer A, Uhlemann R, Reuter G, Humbeck K (2009). Epigenetic programming via histone methylation at *WRKY53* controls leaf senescence in Arabidopsis thaliana. Plant J.

[CR2] Barleben L, Ma X, Koepke J, Peng G, Michel H, Stöckigt J (2005). Expression, purification, crystallization and preliminary X-ray analysis of strictosidine glucosidase, an enzyme initiating biosynthetic pathways to a unique diversity of indole alkaloid skeletons. BBA-Proteins Proteom.

[CR3] Becker W, Apel K (1993). Differences in gene expression between natural and artificially induced leaf senescence. Planta.

[CR4] Brychkova G, Alikulov Z, Fluhr R, Sagi M (2008). A critical role for ureides in dark and senescence-induced purine remobilization is unmasked in the *Atxdh1* Arabidopsis mutant. Plant J.

[CR5] Brychkova G, Xia Z, Yang G, Yesbergenova Z, Zhang Z, Davydov O, Fluhr R, Sagi M (2007). Sulfite oxidase protects plants against sulfur dioxide toxicity. Plant J.

[CR6] Buchanan-Wollaston V (1997). The molecular biology of leaf senescence. J Exp Bot.

[CR7] Chen L, Han Y, Jiang H, Korpelainen H, Li C (2011). Nitrogen nutrient status induces sexual differences in responses to cadmium in Populus yunnanensis. J Exp Bot.

[CR8] Cowan AK, Taylor NJ (2004). Xanthine dehydrogenase and aldehyde oxidase impact plant hormone homeostasis and affect fruit size in ‘Hass’ avocado. J Plant Res.

[CR9] Fageria NK (2007). Yield physiology of Rice. J Plant Nutr.

[CR10] Fujihara S, Yamaguchi M (1978). Effects of allopurinol [4-hydroxypyrazolo (3,4-d)pyrimidine] on the metabolism of allantoin in soybean plants. Plant Physiol.

[CR11] Gan S, Amasino RM (1997). Making sense of senescence (molecular genetic regulation and manipulation of leaf senescence). Plant Physiol.

[CR12] Großkinsky DK, Syaifullah SJ, Roitsch T (2018). Integration of multi-omics techniques and physiological phenotyping within a holistic phenomics approach to study senescence in model and crop plants. J Exp Bot.

[CR13] Guiboileau A, Sormani R, Meyer C, Masclaux-Daubresse C (2010). Senescence and death of plant organs: nutrient recycling and developmental regulation. Cr Biol.

[CR14] Han R, Rasheed A, Wang Y, Wu Z, Tang S, Pan X, Shi Q, Wu Z (2018). Silencing of OsXDH reveals the role of purine metabolism in dark tolerance in rice seedlings. J Integr Agr.

[CR15] Han RC, Wu ZF, Tang SQ, Pan XH, Shi QH, Wang GF, Wu Z (2017). Cloning and expression analysis for xanthine dehydrogenase gene *OsXDH* in rice. J Southern Agric.

[CR16] Hofmann NR (2016). Opposing functions for plant xanthine dehydrogenase in response to powdery mildew infection: production and scavenging of reactive oxygen species. Plant Cell.

[CR17] Inada N, Sakai A, Kuroiwa H, Kuroiwa T (1999). Senescence program in rice (*Oryza sativa* L.) leaves: analysis of the blade of the second leaf at the tissue and cellular levels. Protoplasma.

[CR18] Irani S, Todd CD (2018). Exogenous allantoin increases Arabidopsis seedlings tolerance to NaCl stress and regulates expression of oxidative stress response genes. J Plant Physiol.

[CR19] Jing S, Zhou X, Song Y, Yu D (2009). Heterologous expression of *OsWRKY23* gene enhances pathogen defense and dark-induced leaf senescence in Arabidopsis. Plant Growth Regul.

[CR20] Katalin BN, Omarov RT, Erdei L, Herman LS (2000). Distribution of the Mo-enzymes aldehyde oxidase, xanthine dehydrogenase and nitrate reductase in maize (*Zea mays* L.) nodal roots as affected by nitrogen and salinity. Plant Sci.

[CR21] Khanna-Chopra R (2012). Leaf senescence and abiotic stresses share reactive oxygen species-mediated chloroplast degradation. Protoplasma.

[CR22] Kim J, Shon J, Lee C, Yang W, Yoon Y, Yang W, Yoon Y, Yang WH, Kim YG, Lee BW (2011). Relationship between grain filling duration and leaf senescence of temperate rice under high temperature. Field Crop Res.

[CR23] Knoch E, Dilokpimol A, Geshi N (2014). Arabinogalactan proteins: focus on carbohydrate active enzymes. Front Plant Sci.

[CR24] Lichtenthaler HK, Wellburn AR (1983). Determinations of total carotenoids and chlorophylls a and b of leaf extracts in different solvents. Biochem Soc T.

[CR25] Li Fuzhen, Hu Guocheng, Fu Yaping, Si Huamin, Bai Xuemei, Sun Zongxiu (2005). Genetic analysis and high-resolution mapping of a premature senescence gene Pse(t) in rice (Oryza sativa L.). Genome.

[CR26] Lim PO, Woo HR, Nam HG (2003). Molecular genetics of leaf senescence in Arabidopsis. Trends Plant Sci.

[CR27] Lin CC, Kao CH (2001). Abscisic acid induced changes in cell wall peroxidase activity and hydrogen peroxide level in roots of rice seedlings. Plant Sci.

[CR28] Ma X, Wang W, Bittner F, Schmidt N, Berkey R, Zhang L, King H, Zhang Y, Feng J, Wen Y, Tan L, Li Y, Zhang Q, Deng Z, Xiong X, Xiao S (2016). Dual and opposing roles of xanthine dehydrogenase in defense-associated reactive oxygen species metabolism in Arabidopsis. Plant Cell.

[CR29] Miao Y, Laun T, Zimmermann P, Zentgraf U (2004). Targets of the WRKY53 transcription factor and its role during leaf senescence in Arabidopsis. Plant Mol Biol.

[CR30] Omarov RT, Sagi M, Lips SH (1998). Regulation of aldehyde oxidase and nitrate reductase in roots of barley (*Hordeum vulgare* L.) by nitrogen source and salinity. J Exp Bot.

[CR31] Park SY, Yu JW, Park JS, Li J, Yoo SC, Lee NY, Lee SK, Jeong SW, SeoHS KHJ, Jeon JS, Park YI, Paek NC (2007). The senescence-induced staygreen protein regulates chlorophyll degradation. Plant Cell.

[CR32] Peng XX, Peng S (2000). Degradation of Ribulose-1.5-Bisphosphate carboxylase/Oxygenase in naturally senescing Rice leaves. Acta Phytophysiologica Sinica.

[CR33] Rajinder SD, Pamela P, Trevor AT (1981). Leaf senescence: correlated with increased levels of membrane permeability and lipid peroxidation, and decreased levels of superoxide dismutase and catalase. J Exp Bot.

[CR34] Rogers HJ (2017). Leaf senescence. In: encyclopedia of applied plant sciences.

[CR35] Sagi M, Omarov RT, Lips SH (1998). The Mo-hydroxylases xanthine dehydrogenase and aldehyde oxidase in ryegrass as affected by nitrogen and salinity. Plant Sci.

[CR36] Simpson RJ, Dalling MJ (1981). Nitrogen redistribution during grain growth in wheat (*Triticum aestivum* L.) : III. Enzymology and transport of amino acids from senescing flag leaves. Planta.

[CR37] Tanaka R, Hirashima M, Satoh S, Tanaka A (2003). The Arabidopsis-accelerated cell death gene *ACD1* is involved in oxygenation of pheophorbide a: inhibition of the pheophorbide a oxygenase activity does not lead to the “stay-green” phenotype in Arabidopsis. Plant Cell Physiol.

[CR38] Tang Y, Wen X, Lu C (2005). Differential changes in degradation of chlorophyll-protein complexes of photosystem I and photosystem II during flag leaf senescence of rice. Plant Physiol Bioch.

[CR39] Watanabe S, Kounosu Y, Shimada H, Sakamoto A (2014). Arabidopsis xanthine dehydrogenase mutants defective in purine degradation show a compromised protective response to drought and oxidative stress. Plant Biotechnol-Nar.

[CR40] Watanabe S, Nakagawa A, Izumi S, Shimada H, Sakamoto A (2010). RNA interference-mediated suppression of xanthine dehydrogenase reveals the role of purine metabolism in drought tolerance in Arabidopsis. Febs Lett.

[CR41] Werner AK, Witte C (2011). The biochemistry of nitrogen mobilization: purine ring catabolism. Trends Plant Sci.

[CR42] You S, Zhu B, Wang F, Han H, Sun M, Zhu H, Sun M, Zhu H, Peng R, Yao Q (2017). A Vitis vinifera xanthine dehydrogenase gene, *VvXDH*, enhances salinity tolerance in transgenic Arabidopsis. Plant Biotechnol Rep.

[CR43] Zdunek-Zastocka E, Lips HS (2003). Is xanthine dehydrogenase involved in response of pea plants (*Pisum sativum* L.) to salinity or ammonium treatment?. Acta Physiol Plant.

[CR44] Zhang CJ, Chu HJ, Chen GX, Shi DW, Zuo M, Wang J, Lu CG, Wang P, Chen L (2007). Photosynthetic and biochemical activities in flag leaves of a newly developed super high-yield hybrid rice (*Oryza sativa*. L) and its parents during the reproductive stage. J Plant Res.

[CR45] Zhu L, Yu S, Jin Q (2012). Effects of aerated irrigation on leaf senescence at late growth stage and grain yield of Rice. Rice Sci.

[CR46] Zrenner R, Stitt M, Sonnewald U, Boldt R (2006). Pyrimidine and purine biosynthesis and degradation in plants. Annu Rev Plant Biol.

