# Supplementary material for: Enhancing xanthine dehydrogenase activity is an effective way to delay leaf senescence and increase rice yield
Source: Rice (N Y). 2020 Mar 11;13:16. doi: 10.1186/s12284-020-00375-7 (PMC7065298; doi:10.1186/s12284-020-00375-7)
Supplement: Supplementary file 1 — Additional file 1: Table S1. Primers of vector construction. Table S2. Primers of gene expression analysis. Figure S1. Comparison of panicle length and grain type in wild-type and transgenic lines. (A) Spike shape. 6 (B–D) Grain shape. The white band represents a length of 1cm. Figure S2. Superoxide ion (O2−) levels in wild-type and transgenic lines at different growth stages. Leaves were stained with nitroblue tetrazolium (NBT) for 2 h as described in the Materials and Methods section. The black band represents a length of 100 μm. [file 12284_2020_375_MOESM1_ESM.pdf]

Supplementary Material

Table 1 Primers of vector construction

Primer	Sequence(5'→3')	Restrction site
CDS-F	TCGAGTTGATCGATCGCCCGG A	
CDS-R	ACACGCCATTGGGCTGAGCCCT	
OE-F	TCCGTCGACCTGCAGGTACCCGACAAGCATAAACCTTTAATGACCCT	Kpn I
OE-R	TCCGTCGACCTGCAGGTACCACACGCCATTGGGCTGAGCCCT	Kpn I
1390-F	TGCCTTCATACGCTATTT ATTTGC	
FAD2-R	GAAGCGACGGACCTGGAGAT	
FAD2-F	CCTTTCACAACCTGATTTCCCA	
1390-R	TAATCATCGCAAGACCGGCAACAGG	
Ri1-F	TTCTGCACTAGGTACCAGGCCTGATGGGGTCGCTCACCAGGGC	Kpn I
Ri1-R	CTGACGTAGGGGCGATAGAGCTCTCCTGTTGAAGGGCAGATAGGTCGG	Sac I
Ri2-F	CGGGGATCCGTCGACTACATGGGGTCGCTCCCAGGGC	BamH I
Ri2-R	AGGTGGAAGACGCGTTACTCCTGTTGAAGGGCAGATAGGTCGG	Mlu I

Table 2 Primers of gene expression analysis

Primer	Sequence(5'→3')	gene
Actin-F	ACGGAGCGTGGTTACTCATTC	Actin RT-PCR
Actin-R	ATCGCAACTCAAAAACAACCAG	
XDH-RT-F	ATGGGGTCGCTCACCAGGGC	XDH RT-PCR
XDH-RT-R	TCCTGTTGAAGG GCAGATAGGTCGG	
Ubi2-F	GCTCCGTGGCGGTATCAT	Ubi qPCR
Ubi2-R	CGGCAGTTGACAGCCCTAG	
XDH-qPCR-F	ATGGGGTCGCTCACCAGGGC	XDH qPCR
XDH-qPCR-R	TCCTGTTGAAGGGCGATAGGTCGG	
UO-F	CACCGTGTATGTGAAGGCCA	UO qPCR
UO-R	TTGAACCCGTGGGAATGAGG	

ALN-F	GGGAGGAAGCAGCGATCAAA	ALN qPCR
ALN-R	CGCCAGGTAATGAGGACAGG	
AAH-F	AGTAAGGTACAGCCCGGAGT	AAH qPCR
AAH-R	CACGGCGCAATTTCATTGGA	
WRKY23-F	GAGGAGCGACAACGACATCC	WRKY23 qPCR
WRKY23-R	CGCCCTCGTACGTAGTCAC	
WRKY53-F	GGGTGCCCAAGTTCAAGTCC	WRKY53 qPCR
WRKY53-R	TCCAGTCGTACCTCTGAGCC	
GH27-F	TGACGTGTCTAGTTCCTTTGCT	GH27 qPCR
GH27-R	GAGGAGACTTAGCCATTGACCA	
Pse (t)-F	TGGCACTGATTCATACAGGGAT	Pse (t) qPCR
Pse (t)-R	AAAACAGCAACATCCTGGCAC	
SGR-F	CACGTCCACTGCCACATCTC	SGN2 qPCR
SGR-R	GAGTGGAAGTAGACCCACACC	
ACD1-F	CCAGGCCTTTGCCATTCAAG	ACD1 qPCR
ACD1-R	TGGGGCCATTGGAATGTTGA	
XERO1-F	GAGGGAGGACCACAAGACCG	XERO1 qPCR
XERO1-R	GCCAGTGTTCCCCATCATCT	

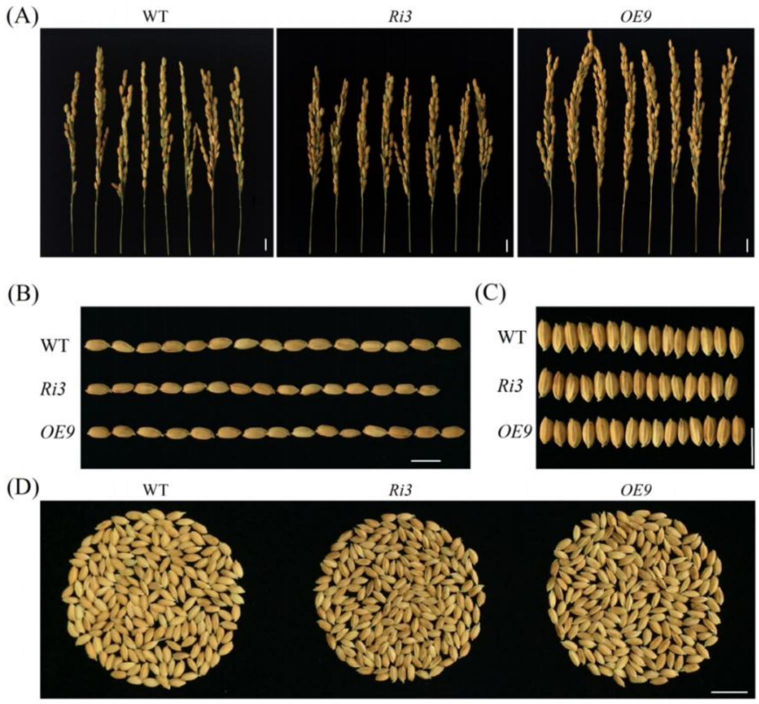

Figure S1 Comparison of panicle length and grain type in wild-type and transgenic lines. (A) Spike shape. (B–D) Grain shape. The white band represents a length of 1cm.

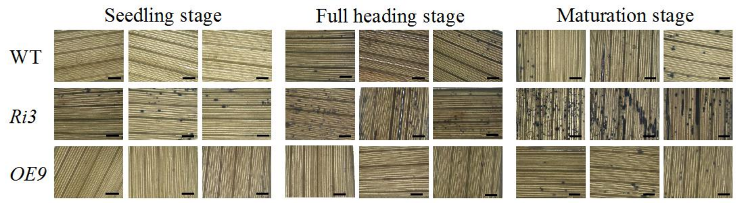

Figure S2 Superoxide ion (O_2^-) levels in wild-type and transgenic lines at different growth stages. Leaves were stained with nitroblue tetrazolium (NBT) for 2 h as described in the Methods section. The black band represents a length of 100 μm.
